# Younger age increases the risk of early prosthesis failure following primary total knee replacement for osteoarthritis: A follow-up study of 32,019 total knee replacements in the Finnish Arthroplasty Register

**DOI:** 10.3109/17453674.2010.501747

**Published:** 2010-07-16

**Authors:** Jaakko Julin, Esa Jämsen, Timo Puolakka, Yrjö T Konttinen, Teemu Moilanen

**Affiliations:** ^1^Medical School, University of Tampere; ^2^Coxa, Hospital for Joint Replacement, Tampere; ^3^Department of Medicine/Internal Medicine, Helsinki University Central Hospital, HelsinkiFinland

## Abstract

**Background and purpose:**

Total knee replacements (TKRs) are being increasingly performed in patients aged ≤ 65 years who often have high physical demands. We investigated the relation between age of the patient and prosthesis survival following primary TKR using nationwide data collected from the Finnish Arthroplasty Register.

**Materials:**

From Jan 1, 1997 through Dec 31, 2003, 32,019 TKRs for primary or secondary osteoarthritis were reported to the Finnish Arthroplasty Register. The TKRs were followed until the end of 2004. During the follow-up, 909 TKRs were revised, 205 (23%) due to infection and 704 for other reasons.

**Results:**

Crude overall implant survival improved with increasing age between the ages of 40 and 80. The 5-year survival rates were 92% and 95% in patients aged ≤ 55 and 56–65 years, respectively, compared to 97% in patients who were > 65 years of age (p < 0.001). The difference was mainly attributable to reasons other than infections. Sex, diagnosis, type of TKR (condylar, constrained, or hinge), use of patellar component, and fixation method were also associated with higher revision rates. However, the differences in prosthesis survival between the age groups ≤ 55, 56–65, and > 65 years remained after adjustment for these factors (p < 0.001).

**Interpretation:**

Young age impairs the prognosis of TKR and is associated with increased revision rates for non-infectious reasons. Diagnosis, sex, type of TKR, use of patellar component, and fixation method partly explain the differences, but the effects of physical activity, patient demands, and obesity on implant survival in younger patients warrant further research.

## Introduction

Young patients suffering from rheumatoid arthritis have been treated with TKR with good long-term results (Lybäck et al. 2000). There have been more cautious indications in younger patients suffering from osteoarthritis because of doubts about compromised prosthesis survival (Rand et al. 2003, Himanen et al. 2005).

In some pioneering studies, the clinical results (Diduch et al. 1997) and the survival of TKR (Diduch et al. 1997, Duffy et al. 1998, Lonner et al. 2000, Tai and Cross 2006) have been good in younger osteoarthritis patients. However, these studies involved low numbers of patients and they were carried out in high-level, specialized centers, which may introduce provider-related bias. In unselected register-based series, young age in general has been associated with a relatively high rate of prosthesis failure (Robertsson et al. 2001, Harrysson et al. 2004, Himanen et al. 2005, Gioe et al. 2007).

Here we present a systematic analysis of the effect of the age of TKR patients on the short-term survival of the prosthesis, using nationwide data from the Finnish Arthroplasty Register. As prosthesis designs and surgical techniques have evolved rapidly with increase in operation volumes since the 1980s, the study was based on operations performed since 1997. Our hypothesis was that young age is associated with impaired prosthesis survival.

## Materials and methods

From Jan 1, 1997 to Dec 31, 2003, 33,223 primary knee replacements performed for primary or secondary osteoarthritis in Finland were recorded in the Finnish Arthroplasty Register. Unicondylar knee replacements (n = 1,204) were excluded, leaving 32,019 TKRs for analysis. Information to the Finnish Arthroplasty Register is recorded prospectively using a specific report form (Puolakka et al. 2001). Reporting of joint replacement operations is obligatory in Finland, and the Finnish Arthroplasty Register covers approximately 96% of primary knee replacements performed in Finland (Jämsen et al. 2009).

The TKRs were followed until Dec 31, 2004 unless death or revision knee replacement occurred before. The mean follow-up time was 3.9 (1–8) years.

### Age groups

Knees were grouped at 5-year intervals based on patient age, starting from 40 years to 80 years, to estimate survival trends using Kaplan-Meier regression analysis. For other analyses, knees were divided into 3 categories by patient age: ≤ 55 years (n = 1,748, 6% of all patients), 56–65 years (n = 6,152, 19%), and > 65 years (n = 24,119, 75%). These cut-off points were used because 55 years has been used in many earlier studies as the cut-off for younger patients (Crowder et al. 2005, Eskelinen et al. 2005, Gioe et al. 2007) whereas 65 years is a common retirement age in several European countries.

### Outcome

The primary outcome was the prosthesis survival rate, which was defined as the proportion of prostheses surviving without revision (removal, exchange, or addition of any prosthesis component) during the follow-up time. Revisions for reasons other than infection were subjected to a separate subgroup analysis.

### Statistics

For comparisons of patient characteristics and operative data between different age groups, chi-squared test or Fisher's exact test was used for categorical variables and analysis of variance was used for continuous variables. Kaplan-Meier survival analysis was used to construct the survival probabilities of TKRs at 1, 3, and 5 years postoperatively.

The comparisons between the age groups ≤ 55 years, 56–65 years, and > 65 years and the analyses of contributory factors were performed using Cox regression analysis. The effect of the following contributory factors was studied: sex, reason for the primary operation (primary or secondary osteoarthritis), TKR type (based on the type of the femoral component: condylar (cruciate-retaining), constrained (including cruciate-substituting designs), or hinge), use of patellar component (installed or not installed), and fixation method (both components cemented, only one component cemented (hybrid fixation), or both components cementless). Finally, the age group and these contributory factors were included in the same Cox regression model. Hazard ratios (HRs) derived from this model are referred to as adjusted hazard ratios. Because the proportional hazards assumption was not met for sex, age group, TKR type, and fixation method, the analyses were run for time intervals ≤ 3.6 years and > 3.6 years (using the median length of follow-up as cut-off point).

All data were analyzed with the knee prosthesis as the statistical unit. It is considered unlikely that the dependency between the knees in bilaterally operated patients would have led to any bias in statistical analyses because bilateral prosthesis failure was extremely rare (n = 24, 12 patients). SPSS software version 15.0 was used for the statistical analyses. Values of p < 0.05 were considered statistically significant.

## Results

### Primary operations

Of the 32,019 TKR operations, 23,051 (72%) were performed on female patients and 8,986 (28%) on male patients. The mean age of the patients was 70 (21–96) years at the time of the primary operation. Primary osteoarthritis was the reason for 31,042 operations (97%) and secondary osteoarthritis was the reason for the remaining 977 operations (3%). The patellar component was installed in 31% of cases (n = 9,801). Of all TKRs, 29,022 (91%) were condylar, 2,799 (9%) were constrained condylar, and 198 had hinge design. Most TKRs, 29,356 (92%), were cemented, 1,846 (6%) were hybrid arthroplasties with either the femoral or the tibial component cemented, and 817 (3%) were cementless. 2,450 TKR operations (8%) were bilateral.

In the younger age groups (≤ 55 years and 56–65 years), the proportions of male patients and patients with secondary osteoarthritis were higher than in the oldest age group. Constrained designs and cementless fixation were slightly more common in the younger age groups. Demographic and perioperative data for the 3 age groups are presented in detail in Table 1.

**Table 1.** Comparison of demographic and perioperative data across the three age groups. Values of p < 0.001 indicate that the 3 age groups differed with respect to each variable tested in univariate analysis

Factor	Category		Age group		p-value
		≤ 55 years (n = 1,748)	56–65 years (n = 6,152)	> 65 years (n = 24,119)	
Mean age	years	51.1 (21–55)	61.4 (56–65)	74.0 (66–96)	–
Gender	female, %	60	67	74	< 0.001 ^**b**^
	male, %	40	33	26	
Diagnosis	primary OA, %	85	96	98	< 0.001 ^**b**^
	secondary OA, %	15	4	2	
Patellar component	not installed, %	66.5	69.4	69.6	0.3
	installed, %	33.5	30.6	30.4	
Prosthesis type	condylar, %	86.6	90.8	90.9	< 0.001 ^**b**^
	constrained, %	12.1	9.0	8.4	
	hinge, %	1.3	0.2	0.7	
Fixation	cemented, %	89.8	90.6	92.1	< 0.001 ^**b**^
	hybrid, %	6.8	6.3	5.6	
	cementless, %	3.4	3.1	2.4	
Laterality	unilateral, %	90.0	89.5	93.2	< 0.001 ^**b**^
	same-day bilateral, %	10.0	10.5	6.8	
Average length of follow-up	years	3.5 (0.0–8.0)	3.8 (0.0–8.0)	3.9 (0.0–8.0)	< 0.001 ^**a**^

OA: osteoarthritis.

^**a**^ p-values from analysis of variance for comparisons between the 3 age groups.

^**b**^ p-values from chi-squared test for comparisons between the 3 age groups.

### Revision operations

During follow-up, 909 (2.8%) of the 32,019 primary TKRs were revised. The mean time until revision was 1.9 years (1 day to 7.8 years). 205 prostheses (23% of all revisions) were revised due to infection. The predominant non-infectious reasons for revision were patellar complications (n = 139), malposition of the prosthesis (n = 109), aseptic loosening (n = 78), and other, unspecified reasons (n = 320), which included revisions due to polyethylene wear and instability (Tamminen et al. 2007). The remaining revisions were performed for periprosthetic fracture (n = 28), dislocation (n = 23), and fracture of the prosthesis (n = 7). Infections accounted for 19%, 15%, and 26% of all revisions among patients aged ≤ 55 years, 56–65 years, and > 65 years, respectively (p = 0.002).

### Prosthesis survival

**Figure 1.**
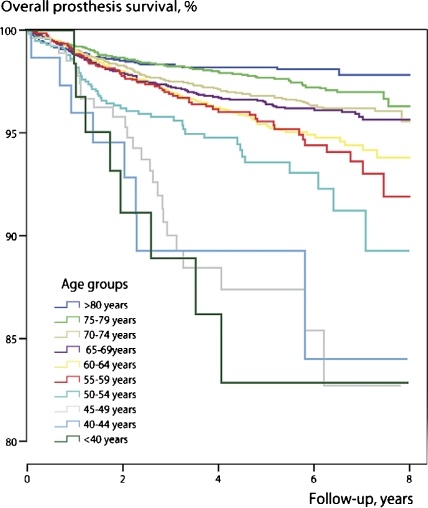
Kaplan-Meier survival curves for different 5-year age groups treated with total knee replacement for osteoarthritis. The endpoint was revision for any reason.

The age of the patient was a strong determinant of prosthesis survival. The overall prosthesis survival improved with increasing age in the 5-year age categories (p < 0.001) (Figure 1). This was also seen in a separate analysis using aseptic failure as endpoint (data not shown). With septic failures, there was no clear correlation between the age of the patient and prosthesis survival (data not shown).

**Figure 2.**
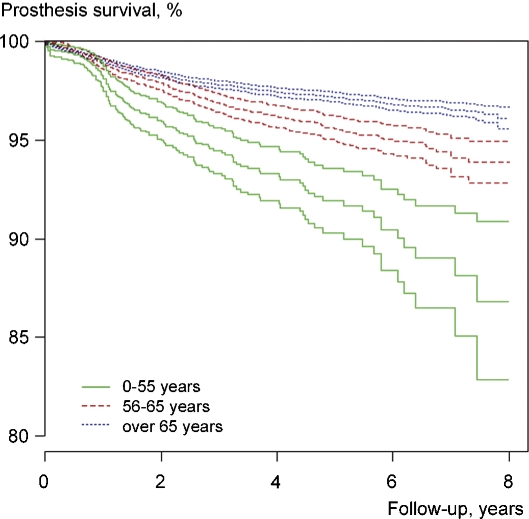
Kaplan-Meier survival curves with their 95% confidence intervals for 32,019 total knee replacements in 3 different age groups. The endpoint was revision for any reason.

**Figure 3.**
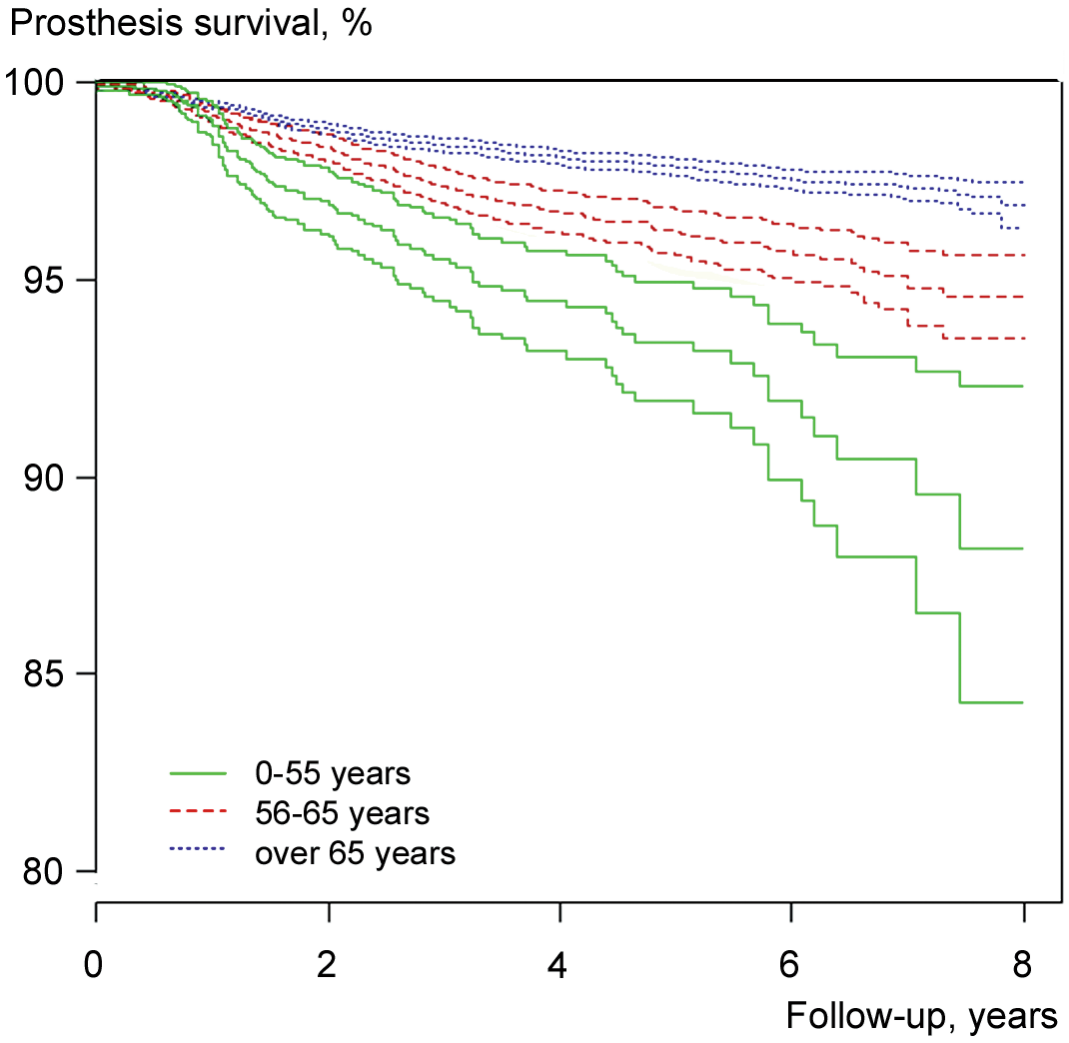
Kaplan-Meier survival curves with their 95% confidence intervals for 32,019 total knee replacements in 3 different age groups. The endpoint was revision for reasons other than infection.

Figures 2 and 3 show the Kaplan-Meier survival curves for the 3 age groups (≤ 55 years, 56–65 years, and > 65 years) with respect to prosthesis failure for any reason and failure for reasons other than infection. At 5 years, TKRs implanted into patients ≤ 55 years showed an overall survival rate of 92% (95% CI: 89.7–93.3). In the 56–65-year-old patients, the corresponding survival rate was 95% (95% CI: 94.8–96.0) and in patients over 65 years it was 97% (95% CI: 96.9–97.3). When only aseptic reasons were considered as endpoints, the respective survival rates were 93% (95% CI: 91.8–95.0), 96% (95.6–96.8), and 98% (97.7–98.1). Compared to patients aged more than 65 years, the risks of prosthesis failure for any reason and for aseptic failure were higher in both younger patient groups during the whole course of follow-up (Table 2). There were no differences in the survival rates for different operation years (data not shown).

**Table 2.** The effect of age group on prosthesis survival, proportion of aseptic failures, and the results of regression analyses

Age group	n (knees)	Prosthesis survival, %			Hazard ratio (95% confidence interval)					
		(95% confidence interval)			Overall risk of prosthesis failure			Risk of prosthesis failure for reasons other than infection		
		At 1 year	At 3 years	At 5 years	Follow-up in total	Follow-up ≤ 3.6 years	Follow-up > 3.6 years	Follow-up in total	Follow-up ≤ 3.6 years	Follow-up > 3.6 years
≤ 55 years	1,748	98 (97.6–98.8)	95 (93.7–95.7)	92 (89.7–93.3)	2.8 (2.3–3.4)	2.1 (1.7–2.7)	5.0 (3.2–8.0)	3.2 (2.5–4.0)	2.5 (1.9–3.2)	4.8 (2.8–8.0)
55–65 years	6,152	99 (98.6–99.0)	97 (96.4–97.2)	95 (94.8–96.0)	1.5 (1.3–1.8)	1.4 (1.2–1.6)	2.0 (1.4–2.9)	1.7 (1.5–2.1)	1.6 (1.3–1.9)	2.0 (1.3–3.0)
> 65 years	24,119	99 (98.8–99.2)	98 (97.5–97.9)	97 (96.9–97.3)	1	1	1	1	1	1

### Predictors of prosthesis survival

Apart from age (the two younger age groups compared to the oldest group), secondary osteoarthritis, lack of a patellar component, and fixation method other than fully cemented fixation were associated with a higher probability of prosthesis failure for any reason and for reasons other than infection in the adjusted analysis during the first 3.6 years of follow-up (Table 3).

**Table 3.** Adjusted hazard ratios (HRs) for revision for any reason and only for reasons other than infection. Adjustments were made for gender, diagnosis, patellar component, TKR type, and fixation method. The values are adjusted hazard ratios (95% CI)

	Adjusted hazard ratio (95% CI)					
	Any reason for revision			Reasons other than infection		
Follow-up in total	Follow-up of ≤ 3.6 years	Follow-up > 3.6 years	Follow-up in total	Follow-up of ≤ 3.6 years	Follow-up > 3.6 years
Age group						
≤ 55 years	2.4 (2.0–3.0)	1.8 (1.4–2.3)	4.6 (2.8–7.4)	2.9 (2.3–3.6)	2.2 (1.7–2.8)	4.5 (2.6–7.8)
56–65 years	1.5 (1.3–1.7)	1.3 (1.1–1.6)	2.0 (1.4–2.9)	1.7 (1.4–2.0)	1.6 (1.3–1.9)	2.0 (1.3–3.0)
> 65 years	1	1	1	1	1	1
Gender						
female	1	1	1	1	1	1
male	1.1 (1.0–1.3)	1.0 (0.9–1.2)	1.0 (0.7–1.4)	1.0 (0.9–1.2)	0.9 (0.8–1.1)	1.1 (0.7–1.6)
Diagnosis						
primary osteoarthritis	1	1	1	1	1	1
secondary osteoarthritis	1.9 (1.5–2.5)	2.5 (1.9–3.3)	1.6 (0.9–3.0)	1.7 (1.3–2.3)	2.3 (1.7–3.3)	1.1 (0.5–2.4)
Patellar component						
installed	1	1	1	1	1	1
not installed	1.2 (1.1–1.4)	1.2 (1.0–1.35)	1.1 (0.8–1.5)	1.4 (1.2–1.7)	1.4 (1.1–1.7)	1.1 (0.7–1.6)
TKR type						
condylar	1	1	1	1	1	1
constrained	1.0 (0.8–1.3)	0.8 (0.6–1.0)	1.0 (0.5–2.2)	0.9 (0.6–1.2)	0.6 (0.4–0.9)	1.1 (0.5–2.6)
hinge	1.5 (0.9–2.8)	1.9 (1.0–3.6)	1.0 (0.2–4.3)	1.8 (0.9–3.4)	2.1 (1.1–4.4)	1.5 (0.4–6.3)
Fixation						
both components cemented	1	1	1	1	1	1
hybrid fixation	1.4 (1.1–1.8)	2.6 (2.0–3.3)	1.2 (0.8–7.4)	1.4 (1.1–1.8)	2.7 (2.0–3.6)	1.1 (0.6–1.9)
both component cementless	1.4 (1.0–1.9)	2.5 (1.7–3.5)	0.8 (0.4–1.9)	1.3 (0.9–1.9)	2.3 (1.5–3.4)	1.0 (0.4–2.3)

The effect of these contributory factors on prosthesis survival was of approximately the same magnitude in different age groups (data not shown), although they were more prevalent in the younger age groups (Table 1). Adjustment for these variables and prosthesis type reduced the overall hazard ratios slightly in the youngest age groups compared to the results of univariate analysis (HR for any revision = 2.4 (95% CI: 2.0–3.0) vs. 2.8 (2.3–3.4); HR for aseptic revision = 2.9 (2.3–3.6) vs. 3.2 (2.5–4.0) (Tables 2 and 3).

There was still a strong association between age group and revision performed for any reason, and for reasons other than infection, in the adjusted analysis also (Table 3).

### Sensitivity analyses

The 3 most frequently used prostheses were Duracon (n = 11,970), AGC v2 (n = 7,690), and PFC Sigma (n = 3,416), which together accounted for 72% of all implants. There were slightly fewer revisions with PFC Sigma (2.2%) than with Duracon and AGC v2 (3.0% and 3.3%, respectively), but the difference did not reach statistical significance in the adjusted analysis.

Antibiotic cement was associated with fewer revisions compared to cemented and hybrid prostheses without antibiotic cement (2.6% vs. 4.9%, adjusted HR = 1.5 (95% CI: 1.3–1.8). Compared to the 7 hospitals with highest volume (> 1,000 operations in 1997–2003), the revision rate for reasons other than infection in the 48 lowest-volume hospitals (< 500 operations) was 1.3-fold increased (95% CI: 1.0–1.5) according to adjusted Cox analysis. Altogether, 76 hospitals performed the operations.

Adjustment for the use of antibiotic cement or hospital volume had no effect on the comparison between the age groups in the adjusted analysis. The results also remained unchanged when the analyses were run in the groups of the 3 most commonly used prostheses (all 3 separately), in the group of prostheses used less than 2,000 times during the observation period, for prostheses fixed with the 2 most commonly used bone cements (Palacos cum gentamycin and Simplex antibiotic), and separately for hospitals with different operation volumes.

Exclusion of patients with secondary osteoarthritis and exclusion of patients who had had both knees operated during the observation period (either simultaneous or staged bilateral operation) (n = 11,010) had no effect on the results of the adjusted analyses (data not shown).

## Discussion

This is by far the most detailed register-based study to systematically compare the survival rates of primary TKR between different age groups in osteoarthritis. In general, our findings support the earlier pioneering register-based studies showing high failure rates in younger patients even with short-term follow-up. In addition, our study demonstrates a clear relationship between the age of the patients in 5-year age categories and the crude (unadjusted) prosthesis survival rates. Patients in the younger age groups were more prone to undergo revision knee replacements for reasons other than infection and the difference between age groups increased during the follow-up.

The patient population consisted of a large and unselected series of primary TKRs performed for osteoarthritis. As a result of obligatory reporting of joint replacement procedures, the Finnish Arthroplasty Register covers approximately 96% of primary knee replacements performed in Finland (Jämsen et al. 2009). Because this study is based on nationwide register data and because emigration from Finland is minor, loss to follow-up is negligible.

Prosthesis survival rates in our study are similar to those in some earlier register-based regional (Gioe et al. 2007) and nationwide studies (Robertsson et al. 2001, Himanen et al. 2005). There were, however, some limitations in these earlier studies: Gioe et al. reported survival rates only for the youngest cohort (75% at 14 years), Robertsson et al. did not differentiate the patients ≤ 55 years from the total cohort of patients aged < 65 years, and in the study by Himanen et al. the analysis was restricted to only one specific prosthesis type.

Compared to the figures reported in register-based series, much better survival rates have been described for young TKR patients in some smaller series. Ritter et al. (2007) reported a survival rate of 97.6% in a 9-year follow-up, and Duffy and co-workers (1998) reported a 99% survival rate in a 10-year follow-up. These survival rates were unadjusted and can probably be explained (at least to some extent) by patient selection and bias arising from the use of specialized clinics. These sources of bias are avoided in nationwide population-based series like ours.

Although secondary osteoarthritis is a relatively rare indication for TKR, it accounted for 15% of operations in patients aged ≤ 55 years and it was found to have a considerable effect on the prognosis of TKRs. The adjusted risk ratio was 1.9 (95% CI: 1.5–2.5) for secondary osteoarthritis compared to primary osteoarthritis. Secondary osteoarthritis has also been found to be a risk factor for revision in another study (Lonner et al. 1999), which suggests that the factors leading to secondary osteoarthritis, e.g. instability, may also strain the prosthetic joint in some patients. On the other hand, this result may also be due to more complicated surgery and possible soft tissue deficiencies related to secondary and posttraumatic osteoarthritis.

Cemented TKR prostheses had somewhat better survival rates than cementless or hybrid prostheses. Gioe et al. (2007) reported similar results, suggesting that cement fixation may improve prosthesis survival in younger patients. In a randomized study in which more elderly patients were included, cemented and cementless prosthesis fixation had a similar 15-year survival (Baker et al. 2007a). Although constrained prostheses are typically used in complex cases with ligamentous incompentence and instability, and considering that using constrained devices increases transmission of forces to the bone-cement interface and thereby predisposes to loosening, constrained TKR designs had short-term survival that was as good as that of condylar designs. This finding was repeated across all age groups, and is in accordance with the results of Lachiewich and Soileau (2006) who demonstrated acceptable survival for constrained designs.

When adjusted for sex, diagnosis, patellar resurfacing, TKR-type, and fixation method—which were also found to be associated with prosthesis failure rate—the hazard ratios for revision in the youngest age group (≤ 55 years) decreased. Hence, the more frequent occurrence of factors associated with poorer prosthesis survival (e.g. male sex and the diagnosis of secondary osteoarthritis) in the younger patients can partly explain the poorer outcomes in the younger age groups. However, the effect of age remained clear even after multiple adjustments and subgroup analyses, which further consolidates the basic observation made in this report. A somewhat similar effect of age has also been reported for total hip replacements (Berry et al. 2002, Mäkelä et al. 2008). It seems that the adjustment for the risk factor profile recorded in the arthroplasty register does not totally explain the differences observed between the 3 age groups analyzed in this work.

Interestingly, reasons for revision other than infection were more common in the 2 younger age groups (81% and 85%) than in the older age group with patients > 65 years of age (74%, p = 0.002). Also, much of the diminution in crude survival rates was explained by the higher demand for revision for reasons other than infection in the younger age groups.

Some factors that contribute to prosthesis revision are not recorded in the Finnish Arthroplasty Register. Young TKR patients may be more prone to need for revision because of their higher functional demands (Iorio et al. 2006). They are also more often dissatisfied with the clinical results of operations (Baker et al. 2007b). Obese TKR patients have poorer survival rates than slim patients (Amin et al. 2006) and obesity has been shown to be more common in younger patients than in older patients who undergo TKR (Changulani et al. 2008). A third important contributing factor that is not usually measured in prosthesis loosening and revision studies is the physical activity of the patients, i.e. steps taken per year (cyclic loading) and engagement in various physical activities, both vocational and sport. Young TKR patients are probably physically more active than the older patients, which may be relevant because it has been suggested that it is not the time in service but the loading of the prosthesis that leads to loosening. Our observation that the risk of prosthesis failure increased during the latter half of the follow-up period (after 3.6 years) supports this view. Also, older patients more often have contraindications for revision, which may lead to underestimation of the true failure rate. In our study, however, a clear difference in survival rates was also observed between the two youngest age groups.

Although register-based studies provide a lot of useful information, they also have their limitations, as discussed above and elsewhere (Paavolainen et al. 1991, Kim et al. 2002, Eskelinen et al. 2005). First of all, the Finnish Arthroplasty Register has not been validated. The reliability of the factors analyzed in this study is, however, probably good as the data on implanted components include serial numbers. The reliability of the diagnoses appears to be acceptable when compared to national Hospital Discharge Register data (Esa Jämsen, unpublished data).

Selection of the main reason for revision from the options provided in the official Arthroplasty Register Reporting Form is not always unambiguous. Moreover, revisions due to polyethylene wear and instability are classified in the group “other, unspecified reasons” and comprise approximately two-thirds of this large group of revisions (Tamminen et al. 2007). Thus, in our study all revisions performed for reasons other than infection were lumped into one category. The deep infections reported most likely represent true cases (Jämsen et al. 2009), but it is acknowledged that some low-grade infections may have been falsely classified as aseptic failures. However, it is improbable that this would have substantially affected the present results.

Some confounding factors not reported in the Finnish Arthroplasty Register, such as functional demand, body mass index, and physical activity may interfere with more detailed data interpretation. Moreover, early prosthesis failure due to patellar complications, instability, and prosthesis malalignment is often a result of poor surgical technique, inappropriate prosthesis selection, or technical errors (Fehring et al. 2001, Mulhall et al. 2006). These provider-related factors could not be analyzed in our study but it is improbable that they would explain the differences between age groups.

In conclusion, in the short-term follow-up the relatively young age of ≤ 55 years was associated with a higher risk of revision, especially for aseptic failure. With increasing age at surgery, the prosthesis survival rates continuously improved. In the category of 56–65 year old patients, the results were already better than in the youngest age group and they were improved futher in patients over 65 years of age. The underlying mechanisms require further investigation, but current knowledge indicates that in patients who are less than 55 years old, total knee replacement should only be used in selected cases when there are no other satisfactory means of giving relief from pain and dysfunction.
